# Validation of a Questionnaire to Assess Patient Satisfaction with an Automated Drug Dispensing System

**DOI:** 10.3390/healthcare12161598

**Published:** 2024-08-12

**Authors:** Palanisamy Amirthalingam, Umar Abdolah Alharbe, Hanad S. S. Almfalh, Saleh F. Alqifari, Ahmed D. Alatawi, Ahmed Aljabri, Mostafa A. Sayed Ali

**Affiliations:** 1 Department of Pharmacy Practice, Faculty of Pharmacy, University of Tabuk, Tabuk 71491, Saudi Arabia; salqifari@ut.edu.sa (S.F.A.); ma-ali@ut.edu.sa (M.A.S.A.); 2 Pharmaceutical Care Department, King Fahad Specialist Hospital, Tabuk 47717, Saudi Arabia; oaa575@gmail.com; 3 Clinical Pharmacy Department, King Khalid Civil Hospital, Tabuk 47915, Saudi Arabia; halmofalh@moh.gov.sa; 4 Department of Clinical Pharmacy, College of Pharmacy, Jouf University, Sakaka 72388, Saudi Arabia; 5 Department of Pharmacy Practice, Faculty of Pharmacy, King Abdulaziz University, Jeddah 21589, Saudi Arabia; amaljabri@kau.edu.sa; 6 Department of Clinical Pharmacy, Faculty of Pharmacy, Assiut University, Assiut 71526, Egypt

**Keywords:** automated drug dispensing system, confirmatory factor analysis, content validity, exploratory factor analysis, reliability statistics, patient satisfaction

## Abstract

Background and objectives: Automated drug dispensing systems (ADDs) have been introduced to improve the efficiency of dispensing and patient safety. The available questionnaires measure patient satisfaction with particular aspects of ADDs. Also, the level of patient satisfaction with ADDs is not widely established. This study aimed to develop and validate a novel questionnaire to assess patient satisfaction with ADDs. Methods: Content and construct validity procedures were used to validate the 20-item questionnaire with four domains, including pharmacy administration, dispensing practice, patient education, and the dispensing system. Two hundred consenting participants took part in this study, from those who visited the outpatient pharmacy in a government hospital. Results: The internal consistency of all four scale items shows acceptable reliability (>0.7). In the exploratory factor analysis, three items were removed due to poor factor loading and cross-loading. In the confirmatory factor analysis, the model has acceptable fit indices, including the comparative fit index (0.937), Tucker–Lewis’s index (0.924), standardized root mean square residual (0.051), root mean square error of approximation (0.057), and χ^2^/df (1.67). The convergent and discriminant validity were established, since the average variance extracted (AVE) was ≥0.5 and the squared correlation (SC) values of one construct with other constructs were less than the AVE of the specific construct. Conclusion: This study offered a reliable and valid 17-item questionnaire incorporating a multi-dimensional four-factor model to evaluate patient satisfaction with ADDs. The validated questionnaire can be utilized to explore patients’ perspectives on ADDs.

## 1. Introduction

Drug dispensing is a fundamental duty of pharmacists; however, there has been an expansion in pharmacy practice services since the transition from technical to patient-centered care services [1]. Drug dispensing is one of the high-risk steps in the medication use process since there is a big chance for medication errors, which might affect patient safety. The American Society of Health-System Pharmacists endorsed the use of automated drug dispensing systems (ADDs), leading to a substantial reduction in the workload of pharmacists, allowing them to focus on patient care and improving patient safety by minimizing medication errors [2,3,4,5]. The performance of hospital pharmacies that adopted ADDs increased, in terms of prescription filling, counting accuracy, safety, and adherence. Also, ADDs minimize delays in drug supply, cost, and stock outages [6].

Utilizing ADDs, the pharmacist has the potential to enhance the clinical care of patients due to a reduction in workload and time during the dispensing process [7]. However, these advantages of ADDs were not fully transferred to improve patient care, as shown in a recent study that concluded that the time spent by the pharmacist for patient education in ADDs was still comparable with traditional drug dispensing systems (TDDs) [3]. Pharmacists need the motivation to effectively utilize their free time by expanding their role in reviewing medication use, optimizing medication administration records, and improving patient care [3,8]. Although ADDs are effective, human error might harm patient safety due to the failure of interface points between the ADD’s components [9].

The implementation of ADDs decreased medication errors; however, it has not reduced all errors, and there was no significant impact on patient safety [5]. A recent study addressed that the chance of error in ADDs ranges from 0.12 to 8.99 (95% confidence interval) with an odds ratio (OR) of 1.03. Also, they added that the relative risk (RR) of the occurrence of errors increases by more than 700% in the worst-case scenario in ADDs [10].

The effective implementation of ADDs was independent of hospital management; hence, monitoring pharmacist skills in ADDs and obtaining patient satisfaction periodically can improve patient safety by ruling out the pitfalls during the adoption of ADDs [3,11]. Although Bardage et al. attempted to understand the patient perspectives on ADDs, patient satisfaction has yet to be assessed with a structured validated questionnaire [12]. Hence, the present study aimed to develop and validate a structured questionnaire to investigate patient satisfaction with ADDs.

## 2. Materials and Methods

### 2.1. Study Design

This study was conducted from 1 February 2023 to 31 July 2023 at the Governmental Hospital in Tabuk, Saudi Arabia. A new questionnaire was designed, partially adopting some items from the previously developed questionnaire by Ismail et al. (2020), which measures patient satisfaction with pharmacy services in public health clinics [13]. The required permission from the corresponding author was granted via email. The new 20-item questionnaire was tested for content validity and construct validity.

### 2.2. Sample Size and Sampling Method

The sample size was calculated using a 1:10 ratio (number of items: participants) to ensure the model’s validity and reliability [14]. Since the questionnaire consisted of 20 items, we included 200 patients who already utilized the pharmacy services directly or on behalf of their family members and friends at the study site. This study used a convenient sampling method. The patients were requested to participate in the survey after obtaining medications from the outpatient pharmacy.

### 2.3. Ethical Approval and Informed Consent

This study was approved by the Institutional Review Board, Ministry of Health, Tabuk, Saudi Arabia (Reference number: TU-077/023/182). Before including the patient or patient’s representatives in the study, written informed consent was obtained from them.

### 2.4. Details of the Questionnaire

The questionnaire has two parts. The first one consists of the characteristics of the study participants (Table 1). The second part has four different domains (Table 2), addressing patient satisfaction with pharmacy administration (Part I), dispensing practice (Part II), patient education (Part III), and dispensing system (Part IV). The questionnaire utilized a 5-point Likert scale, with each item rated on a scale from ‘strongly disagree’ to ‘strongly agree’ (ranging from 1 to 5). The questionnaire was structured in the English language by the two faculty members in the Department of Pharmacy Practice and reviewed by four other faculty members for appropriateness to assess patient satisfaction regarding simplicity, suitability, sentence structure, and ambiguity.

### 2.5. Content Validity

Five experts in pharmacy practice were recruited from other institutions to validate the content [14]. Item-level content validity indexes (I-CVIs) were used to determine the relevance of items and the averaging of scale-level content validity indexes (S-CVI/Ave) for the overall questionnaire. Scores of I-CVIs ≥ 0.78 and S-CVI/Ave ≥ 0.90 were considered excellent content validity [14,15,16].

### 2.6. Construct Validity

Internal consistency was used to measure the reliability and reproducibility of the scores of the questionnaires by assessing Cronbach’s α and McDonald’s ω coefficients. Cronbach’s α and McDonald’s ω coefficients of values >0.9, >0.7 to ≤0.9, and <0.7 are considered excellent, good, and poor, respectively [17,18].

The model was constructed with a four-factor structure, and each factor had five items initially. Exploratory factor analysis (EFA) used the maximum likelihood extraction with a Varimax rotation method [19]. The inclusion criteria involved factor loading >0.5 to retain the corresponding items under their respective factors [20,21]. Bartlett’s test for sphericity of <0.05 and the Kaiser–Meyer–Olkin—Measuring Sampling Adequacy (KMO-MSA) value ≥ 0.7 were considered acceptable for sampling adequacy [22,23]. The threshold for the cumulative percentage of variance was 50.2% and the acceptable cut-off value of commonalities was >0.25 [24,25].

In confirmatory factor analysis (CFA), the robust unweight least square estimation method was used since the ordinal data were used to construct the model [26,27]. The model fit was established using more than three fit indices by following the recommendations of Hair et al., 2010 [28]. The Chi-square *p*-value was > 0.05, along with a Chi-square to degrees of freedom ratio (χ2/pdf) of less than 5, of a good model fit. The other fit indices, including root, mean square error of approximation (RMSEA) ≤ 0.08, standardized root mean square residual (SRMR) ≤ 0.08, comparative fit index (CFI) > 0.9, and Tucker–Lewis index (TLI) > 0.9, were considered as good model fit [29]. Structural equation modeling interpretation was performed by following the checklist [30]. The average variance extracted (AVE) of ≥ 0.5, construct reliability for the latent factors (≥0.7), and standardized factor loadings (>0.7) were considered as satisfying the convergent validity [19]. The squared correlation (SC) values of one construct with other constructs are less than the AVE of a specific construct, and the factor correlation matrix < 1 for the factors reveals that the two factors not explaining the same dimension were considered as satisfied discriminant validity [19,31,32].

### 2.7. Statistical Analysis

Reliability statistics and factor analysis were used to validate the questionnaire since recent studies have mostly adopted these methods [13,19]. This was performed using Jeffreys’s Amazing Statistics Program (JASP).

## 3. Results

### 3.1. Characteristics of the Study Participants

A total of 200 participants were involved in validating the questionnaire (Table 1). Female participants predominantly consented to participate in the study, and 73.5% of the participants were 18–30 years of age. In total, 76% of the study participants were graduates, and 23% had at least completed their school education. Employment status revealed that the majority (39%) of the study participants worked in private companies. Family revenue was less than SAR 5000 among 68.5% of the study participants. Also, 77.5% of the study participants visited the hospital for acute care regarding their minor ailments, while the remaining 22.5% visited to manage chronic illness. Predominantly, the study participants (63%) resided in Tabuk City, and the remaining participants were from outside Tabuk City. Most of them were single (69%), followed by married participants (28.5%).

### 3.2. Content Validity

The I-CVIs for the relevancy of the questionnaire ranged from 0.8 to 1 and the S-CVI/Ave was >0.9. Therefore, the 20-item questionnaire with four different domains demonstrated excellent content validity.

### 3.3. Internal Consistency of the Questionnaire

Each section of the questionnaire exhibited acceptable internal consistency with Cronbach’s α and McDonald’s ω coefficients > 0.7, indicating acceptable reliability (Table 2).

### 3.4. Exploratory Factor Analysis

The KMO-MSA (0.857) and Bartlett’s test for sphericity (*p* < 0.001) indicated that the factor analysis has an acceptable sample size. The total cumulative percentage of variance (50.8%) and commonalities of all items > 0.25 revealed that the proportion of variance explained by the factors was satisfactory. Three items had factor loading < 0.5 or cross-loading > 0.32 and were therefore removed from the questionnaire. Item 5 in factor 1 with a factor loading = 0.479, followed by item 6 and item 9 with cross-loading in two factors, were removed from the questionnaire (Table 2).

### 3.5. Confirmatory Factor Analysis

**Figure 1:**
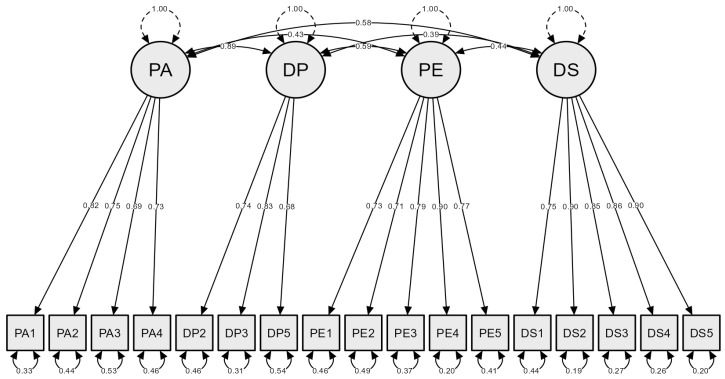
Confirmatory factor analysis of a four-factor model with 17 items.

The confirmatory factor analysis examined the four-factor model with the 17-item questionnaire proposed by the EFA for the model’s fitness towards assessing patient satisfaction with automated drug dispensing systems. The four-factor had acceptable model fit indices, including CFI (0.937), TLI (0.924), SRMR (0.051), RMSEA (0.057), and χ^2^/df (1.67), illustrated in Table 3. Also, all the factors satisfied a composite reliability (>0.8). The convergent and discriminant validity were established since the AVE (≥0.5), and SC values of one construct with other constructs are less than the AVE of the specific construct (Table 4). The standardized factor loadings and factor correlations are represented in Figure 1. Only two items had factor loadings close to 0.7 (PA3 and DP5), and the remaining items had considerable loadings (>0.7). The factor correlations ranged between 0.39 (Dispensing Practice ↔ Dispensing system) and 0.89 (Pharmacy administration ↔ Dispensing Practice). None of these values are close to 1, which indicates that the factors did not represent a similar dimension for the constructed CFA model (Figure 1).

## 4. Discussion

The study designed and validated a new questionnaire for evaluating patient satisfaction with ADDs. The questionnaire may also be used to assess patient satisfaction with other types of dispensing systems. In CFA, the model fit indices, convergent, and discriminant validity were used to investigate the suitability of the model for assessing the patient perceptions of ADDs. The five model fit indices CFI, TLI, RMSEA, SRMR, and χ^2^/df, supported the model fitness, as Hair et al., 2010 quoted that more than three satisfied fit indices were required for a reputable model [27].

This study also assessed the sub-types of construct validity, including convergent and discriminant validity. Standardized factor loadings, construct reliability, and AVE explained convergent validity. The standardized factor loadings in PA3 (0.69) and DP5 (0.68) remained reasonable since the previous researchers established their model with values close to 0.7 [19,33]. The remaining fifteen items in our model have appreciable factor loadings (>0.7). This study revealed a satisfactory construct reliability of all the factors with values of ≥0.7 in the four-factor model [19]. An AVE of >0.5 also emphasized the convergent validity of the model [27,28]. None of the factor correlations were close to 1 (Figure 1) and the AVE exceeded SC (Table 3), which means that the latent factors had no relationship with each other, affirming the model’s discriminant validity. Therefore, the 17-item four-factor model questionnaire is suitable for assessing patient satisfaction with ADDs [29,34].

Initially, the content validity was established in a four-factor model with 20 items, where each factor had 5 items. I-CVIs and S-CVI/Ave were used to assess the content validity. The experts for content validity were chosen from various institutions to rule out possible bias in selection. The results of I-CVIs and S-CVI/Ave offer sufficient evidence to move forward for the EFA [14,15].

All four factors had acceptable internal consistency (>0.7) in both Cronbach’s α and McDonald’s ω reliability statistics [16,18]. This study had a sample size of 200 for validation, which was found to be adequate since KMO-MSA (0.857) and Bartlett’s test for sphericity (*p* < 0.001) reject the null hypothesis of the identical correlation matrix [19]. Hence, the EFA began data extraction. The item had factor loadings < 0.5 (PA5), and two other cross-loaded items (DP1 and DP4) were removed from the questionnaire [20]. On the other hand, the cumulative variance for the four-factor model was 50.8, which was higher than the threshold value [25]. Therefore, the amount of variance explained by the factors was satisfactory.

Patient satisfaction with pharmaceutical care services, outpatient pharmacy facilities, ambulatory care pharmacy services, and electronic health records has already been investigated in Saudi Arabia [35,36,37]. Pharmacist perception of ADDs was recently established in Saudi Arabia [3]. In this context, the present study pioneered the validation of a questionnaire to explore patient perceptions towards ADDs. Bardage and Ring, 2016, investigated patient perspectives on a single domain of ADDs in multi-dose dispensing [12]. Hence, the present study offers a validated questionnaire to assess patient satisfaction with four domains. Each factor in this questionnaire has a different dimension; therefore, this multi-dimensional questionnaire can assess patient satisfaction in various aspects.

### Strength and Limitations

Sample size remains controversial when validating a questionnaire. We included a sample size of 200 according to the ratio 1:10 (item/number of participants) mentioned in the methods. However, the KMO sampling adequacy and Bartlett’s test were satisfactory in constructing the four-factor model. The sample may not be representative of the general population since most of the participants belong to the 18–30 age group and were graduates. Also, the questionnaire was developed in English, not in Arabic, which might have led to a language bias. Also, the questionnaire cannot be generalized since pharmacies in different countries have greater variances regarding the services offered to the patients.

## 5. Conclusions

This study offered a reliable and valid 17-item questionnaire incorporating a multidimensional four-factor model to evaluate patient satisfaction with ADDs. The validated questionnaire can be utilized to explore patients’ perspectives on other dispensing systems.

## Figures and Tables

**Table 1:** Characteristics of the study participants.

Characteristics	N (200)	%
Gender		
Male	88	44
Female	112	66
Age		
18–30	147	73.5
31–50	43	21.5
51–64	9	4.5
More than 65	1	0.5
Residence		
Tabuk city	126	63
Outside Tabuk city	74	37
Education		
Graduates	152	76
Higher secondary school	26	13
Secondary school	17	8.5
Primary school	3	1.5
Illiterate	2	1
Marital status		
Single	138	69
Married	57	28.5
Divorcee	3	1.5
Widow	2	1
Employment status		
Private	78	39
Government	58	29
Housewife	50	25
Business	12	6
Students	2	1
Family revenue (in Saudi Riyal)		
<5000	117	68.5
5000 to 10,000	62	31
>10,000	21	10.5
Type of care		
Acute care	155	77.5
Chronic care	45	22.5

**Table 2:** Factor loadings, communalities, percentage of variance, and reliability statistics.

Factor	Item No.	Item	Factor Loadings				Communalities
			Factor 1 *	Factor 2 **	Factor 3 ***	Factor 4 ****	
Factor 1 *	PA 1	Maintenance of the pharmacy is good	0.596	−0.194	0.215	0.008	0.439
	PA 2	The pharmacy area is neat and hygienic	0.616	0.181	0.014	0.061	0.416
	PA 3	The Pharmacy has sufficient space for the drug dispensing process	0.792	0.244	0.025	0.068	0.692
	PA 4	A sufficient number of pharmacists working in pharmacy	0.707	0.063	0.118	0.061	0.521
	PA 5	Drugs are readily available for dispensing	0.479	−0.261	0.102	0.052	0.310
Factor 2 **	DP 1	The Pharmacist more actively participated in the medication dispensing	0.036	0.701	0.324	0.071	0.603
	DP 2	The Pharmacist verifies all the medications before dispensing	0.008	0.673	0.285	0.124	0.549
	DP 3	The medication label is clear and understandable	−0.048	0.771	0.259	0.044	0.666
	DP 4	The Pharmacist is able to dispense the medications quickly	0.014	0.445	0.218	0.496	0.492
	DP 5	The Pharmacist dispensed all the medications in the correct quantity	−0.124	0.624	0.301	−0.109	0.508
Factor 3 ***	PE 1	The Pharmacist explains how to take medications	−0.062	0.239	0.546	0.128	0.376
	PE 2	The Pharmacist explains the side effects of medications	0.100	0.236	0.553	0.089	0.380
	PE 3	The Pharmacist provides all the information that I need	−0.004	0.292	0.586	0.142	0.448
	PE 4	The Pharmacist always listen to me and clear my doubts	0.104	0.181	0.728	0.140	0.593
	PE 5	The Pharmacist ensures that I fully understand the explanation given	−0.026	0.143	0.726	0.058	0.552
Factor 4 ****	DS 1	This drug dispensing system is more efficient	−0.021	−0.035	−0.005	0.589	0.348
	DS 2	The drug dispensing system is safe for the patient	0.040	0.001	0.016	0.781	0.612
	DS 3	This dispensing system reduces patient time	0.079	−0.028	0.068	0.774	0.611
	DS 4	This dispensing system reduces pharmacists’ time	0.043	−0.031	0.040	0.711	0.510
	DS 5	This dispensing is system useful to the hospital	0.102	0.009	−0.030	0.722	0.533
		Cronbach’s α	0.776	0.829	0.805	0.840	
		McDonald’s ω	0.780	0.836	0.808	0.842	
		Percentage of variance	13.2	12.9	12.4	12.3	
		Cumulative percentage of variance	13.2	26.1	38.5	50.8	

* Factor 1—Pharmacy Administration (PA); ** Factor 2—Dispensing Practice (DP); *** Factor 3—Patient Education (PE); **** Factor 4—Dispensing System (DS); Items in red colored font—Factor loading < 0.5 or Items with cross-loading > 0.32.

**Table 3:** Model fit indices of CFA.

	CFI	TLI	SRMR	RMSEA	90% Confidence Interval		χ^2^	df	χ^2^/df
					Lower	Upper			
Observed	0.937	0.924	0.0519	0.057	0.051	0.072	189	113	1.67
Reference	>0.9	>0.9	<0.08	<0.08	-	-	-	-	<6

CFI: Comparative Fit Index; TLI: Tucker–Lewis Index; SRMR: Standardized Root Mean Square Residual; RMSEA: Mean Square Error of Approximation; χ^2^: Chi-Square; df: Degrees of Freedom.

**Table 4:** Convergent validity and discriminant validity of CFA.

	Construct Reliability	Factor 1 *	Factor 2 **	Factor 3 ***	Factor 4 ****
Factor 1 *	0.852	**0.594**			
Factor 2 **	0.813	(0.046)	**0.593**		
Factor 3 ***	0.842	(0.124)	(0.279)	**0.520**	
Factor 4 ****	0.885	(0.047)	(0.369)	(0.093)	**0.607**

* Factor 1—Pharmacy Administration; ** Factor 2—Dispensing Practice; *** Factor 3—Patient education; **** Factor 4—Dispensing Practice; Average Variance Extracted (AVE) in bold letters; Squared Correlations (SC) mentioned in bracket; AVE > 0.5 and AVE > SC values (for the corresponding factors) determine the convergent and discriminant validity

## Data Availability

Data are not publicly available; however, they may be available to the corresponding author upon request.
